# Central Obesity as a Major Determinant of Body Image Concerns: A Comparative Study Between Egyptian and Lebanese University Females

**DOI:** 10.1155/jnme/8152494

**Published:** 2025-06-21

**Authors:** Germine El-Kassas, Nour Kabbara, Fouad Ziade

**Affiliations:** ^1^Department of Technology of Nutrition and Food Safety, Faculty of Applied Health Sciences Technology, October 6 University, Giza, Egypt; ^2^Nutrition and Dietetics Department, Faculty of Health Sciences, Beirut Arab University, Tripoli, Lebanon; ^3^Faculty of Public Health, Lebanese University, Tripoli, Lebanon

**Keywords:** body image, dieting, obesity, weight perception

## Abstract

**Background:** Body image is a multidimensional construct influenced by a myriad of psychosocial and lifestyle factors. The present study has been conducted to explore the prevalence of body image concerns and its associated determinants among female Egyptian and Lebanese university students.

**Methods:** Through a cross-sectional comparative study, a sample of 634 females was recruited from two Egyptian and Lebanese universities. Data were collected using an interview questionnaire to assess the various sociodemographic characteristics, lifestyle behaviors, dietary factors, and perceived weight status. The existence of body image concern was evaluated using the validated short form of the Body Shape Questionnaire (BSQ-16).

**Results:** The present data unveiled a relatively alarming prevalence of body image concerns, 46.7.8% and 48%, among the Egyptian and Lebanese university females, respectively, with a statistically significant difference between the 2 studied groups with regard to the degree of body image concern (*p*=0 − 009). The results of regression analysis declared that enlarged waist circumference was the strongest significant determinant of body image concerns (*t*-test *p* value = 0.000 and 0.001 among Egyptian and Lebanese university females, respectively). Adopting dieting practices (*t*-test *p* value = 0.000 and 0.001) and parental obesity (*t*-test *p* value = 0.02 & 0.002) were significantly associated with higher body image concerns' scores among Egyptian and Lebanese university females, respectively. Distinctively, perceived body image (*t*-test *p* value = 0.000), meal pattern (*t*-test *p* value = 0.004), and employment status (*t*-test *p* value = 0.002), were significantly associated with higher body image concerns in the Egyptian group only.

**Conclusions:** The study findings call for tailored, culture-specific intervention programs that enable students to improve their self-acceptance and lead a healthy life.

## 1. Introduction

Body image concern is an emerging significant public health issue in both developed and developing countries [1, 2]. The concept of body image was first introduced nearly a century ago by Schilder who stated that the mental images that individuals have of their bodies explain how they are introduced to them [3]. There has been a growing research interest in the understanding of the body image concept in Western countries throughout the past decade [3, 4]. Body image is now recognized as a multidimensional concept that encompasses one's beliefs about appearance, feelings about body size and shape, as well as perceptions and sense of embodiment [3].

Substantial research evidence indicated that negative body image is linked to low self-esteem, poor mental well-being, eating disorders, and depression [5–8]. Moreover, available literature suggested that lower body image satisfaction appears to increase the chances of engaging in unhealthy weight control behaviors such as crash dieting and the use of extreme weight control measures [9, 10] and are more prone to accept cosmetic surgeries [11, 12]. Such weight loss behaviors will likely result in weight gain and poorer overall health [12, 13].

Body image is influenced by various factors including social, cultural, biological, historical, psychological, and personal factors [14, 15]. Several studies have investigated the sociodemographic determinants of body image concerns or dissatisfaction among female university students, which resulted in conflicting conclusions. Some authors reported significant associations between some sociodemographic determinants such as age and income and body image perceptions [16, 17]. In contrast, several studies had failed to find any associations with any of the sociodemographic variables including age, parental education, marital status, type of studied course, employment, living arrangement, study shift, and income [18, 19]. Furthermore, few studies have examined the associations between body image dissatisfaction and health-related behaviors such as physical inactivity and smoking among university students and revealed some discrepancies in these associations [20]. This in turn indicates the need for further research to explore predictors of body image concerns and dissatisfaction in conjunction with other factors suggested to be influencing body image satisfaction.

Accumulating research in Western countries has documented that overweight and obese individuals are more prone to body image dissatisfaction compared to those of normal weight [21, 22]. However, none of the reported studies was able to provide clear evidence that increased body weight causes lower body satisfaction and this could be attributed to methodological issues used in those studies [23]. Some authors suggested that an increase in body weight is associated with social pressure to lose weight resulting in a reduction in body satisfaction [24]. Inconsistently, other authors concluded that low body satisfaction may lead to unhealthy dieting behaviors, which lead to weight gain later on [25].

Attaining and maintaining a healthy body image among youth is now considered a challenge, especially among young females [15]. This has been shown as progressively rising prevalence rates of body image concerns and dissatisfaction among the university female population [26, 27]. Researchers concluded that intense physical and behavioral changes during adolescence combined with the psychosocial pressures of university life might increase the vulnerability to many health risks including body image concerns and dissatisfaction [28]. Available literature suggested also that body image concerns and dissatisfactions are rising among female university students due to the increasing pressure of peers and media to achieve a slim body shape [16]. Over the past years, many developing countries including Egypt and Lebanon have been facing the effects of globalization, which resulted in several changes in cultural beliefs [31]. This in turn has been reflected in the adoption of Westernized lifestyles such as low intake of fruits and vegetables and high intake of fast foods, smoking, and low physical activity [30, 31]. In addition, this Westernized lifestyle has been coupled with changing norms such as the desire to be thin which may predispose to body image concerns and dissatisfaction [32, 34].

Throughout the past few years, both the Lebanese and the Egyptian communities have suffered exposure to the COVID-19 pandemic and economic crises. These crises ultimately resulted in more social media engagement and aggravation of stressors among the highly vulnerable female university students [33, 34]. There is a scarcity of data concerning body image determinants in developing Arab countries that have experienced many socioeconomic and developmental changes during the past few years. On the basis of these premises and given the detrimental outcomes associated with poor body image and body image concerns, the present study was designed aiming at the exploration of associated correlates of body image concerns among female university students in Egypt and Lebanon through a comparative approach. This information in turn may serve as the basis for tailoring the most relevant culture-specific preventive programs within the context of an extended life cycle approach. These programs could aid in the health promotion and well-being of this important sector of the young generation and future mothers and their families, which could have a positive impact on the community as a whole later on.

## 2. Methods

### 2.1. Study Design and Participants

Through a cross-sectional comparative study design, a survey was conducted on a sample derived from the study population defined as the students at two private universities in Egypt and Lebanon, namely, October 6 University (O6U) and Beirut Arab University (BAU). O6U is located in the 6th of October city/Giza governorate which receives students originating out of different governorates from all over Egypt. The three campuses of BAU located in the capital (Beirut), North Lebanon (Tripoli), and Chouf (Debbeih) were included in the survey. These three campuses are located in the three major governorates of Lebanon. Participants from both countries were female students from lower-middle and higher-middle social classes, which represent the majority of university students in both communities.

#### 2.1.1. Sampling Procedure

**Figure 1:**
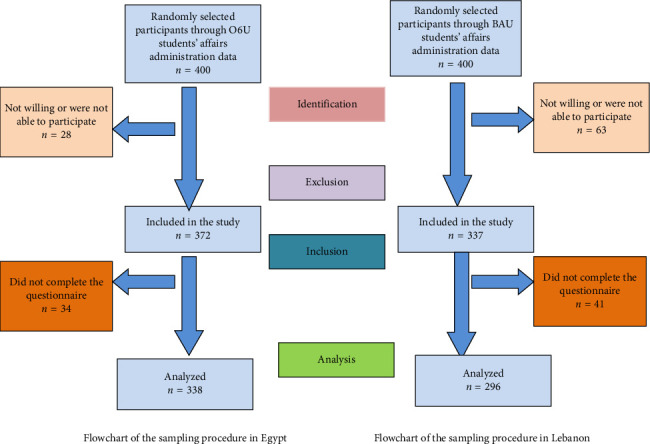
Sampling plan.

A stratified proportionate random sample of females (18–25 years) with strata being the different faculties at O6U and BAU was employed for the purpose of this study. A total sample size of 580 participants (290 in each country) was determined to be adequate to achieve 80% statistical power at a 5% significance level. As shown in Figure 1, the sampling plan included a random selection of 400 students from each of O6U and BAU students' affairs departments' data depending on the calculated sampling strata from the different faculties. Students who refused the invitation to participate in the study for personal reasons or those who were unable to participate due to time constraints or due to local instabilities and poor security issues in the Lebanese group were excluded.

The inclusion and exclusion criteria were strictly applied during the recruitment process. The inclusion criteria implied being a female within the age group of 18–25 years who regularly attends the university and expressed interest to participate in the study. The exclusion criteria were any student having any chronic disease that may affect the metabolism such as chronic kidney or liver diseases and diabetes mellitus, students suffering from physical motor disability, and those on regular intake of specific drugs that may affect appetite or weight control. Participants were provided with a brief explanation about the purpose of the study and guarantee of anonymity and all gave their informed female students: 338 from Egypt and 296 from Lebanon. The study was conducted in accordance with the Declaration of Helsinki, and the protocol was approved by the Scientific Ethics Committee of O6U and the Institutional Review Board of BAU.

#### 2.1.2. Data Collection

Based on the previously published instruments used among the university students, a structured anonymous one-to-one interview pretested questionnaire was developed by the authors [30]. To standardize data collection procedures, all interviewers participated in a specialized training conducted by the authors prior to the study. During free time, the interview questionnaires were applied to each participant separately to ensure full privacy. Data collection was under the continuous supervision of the authors. The questionnaire retrieved information about the sociodemographic characteristics, lifestyle behaviors, dieting attempts, meal patterns and dietary intake, physical activity levels, and body image concerns followed by anthropometric measurements.

### 2.2. Measures

#### 2.2.1. General and Sociodemographic Characteristics

Questions inquiring about age, gender, marital status, type of major of study, the type of current residence, educational level of parents, and crowding index were asked to define the general and sociodemographic characteristics of the study sample.

The evaluation of the influence of the social and health norms was performed by asking the question “What motivates you to eat healthy?,” which received 4 responses which are as follows: parents, peers, keeping good health, and weight control, which were then regrouped into two categories: social (for motivation by peers and parents) and health concerns (for motivation by attaining good health and weight control).

#### 2.2.2. Anthropometric Assessment

Anthropometric assessment including weight, height, and waist circumference (WC) measurements were taken by trained researchers using standardized methods [35] and regularly calibrated scales. Standing height with bare feet was measured to the nearest 0.1 cm using a stadiometer. Participants, dressed in light clothing, were weighed to the nearest 0.1 kg. Body mass index (BMI) was calculated using the formula: body weight (kg)/height (m^2^), and categorized according to the World Health Organization (WHO) criteria for overweight and obesity classification [36]. BMI values were divided into four categories: underweight (BMI ≤ 18.5 kg/m^2^), normal weight (BMI between 18.5 and 24.9 kg/m^2^), overweight (BMI between 25 and 29.9 kg/m^2^), and obese (≥ 30 kg/m^2^) [38]. To identify subjects with an increased risk of metabolic complications, the WHO cut-off point for WC was used, with values greater than 80 cm for females [37].

#### 2.2.3. Dietary Assessment

The meal pattern of the study participants was assessed by inquiring about the number of daily meals, snacks, and regular breakfast consumption. Previous or current dieting habits were evaluated by asking participants whether they had ever tried to lose weight, using the following questions: “Have you ever been on a weight loss diet?” and “Did you follow a diet last year?.”

#### 2.2.4. Physical Activity and Lifestyle Variables

To evaluate the students' physical activity levels, the short form of the International Physical Activity Questionnaire (IPAQ), assessing the previous 7 days (IPAQ-S7S), was utilized [38]. We adhered to the guidelines provided in the IPAQ manual to ensure reliability and validity. The IPAQ short form evaluates three specific activity types: leisure-time activities, work-related and transport-related activities, and domestic tasks. These activities include walking, moderate-intensity exercises, and vigorous-intensity exercises. For each specific type of activity, the frequency (in days per week) and duration (in minutes per day) were recorded separately. The above items were designed to generate separate scores for walking and moderate-intensity and vigorous-intensity activities, along with a total score representing overall activity levels. To calculate the total score, the duration (in minutes) and frequency (in days) of walking and moderate-intensity and vigorous-intensity activities were summed. Physical activity levels (as per the short form) were classified into low, moderate, or high categories according to the official IPAQ scoring protocol [39].

Smoking status was categorized according to WHO criteria [42]. Smokers were identified as individuals who, at the time of the study, smoked either daily (at least one cigarette per day) or occasionally (fewer than one cigarette per day). Experimenters and those currently smoking during the survey but who had smoked fewer than 100 cigarettes in their lifetime were also categorized as smokers. Nonsmokers were individuals who did not smoke at the time of the survey, including both ex-smokers and never-smokers. Ex-smokers were individuals who had previously smoked but had quit entirely, while never-smokers were individuals who had never smoked or had smoked fewer than 100 cigarettes in their lifetime and were not smoking at the time of the survey or in the month preceding it.

#### 2.2.5. Assessment of Body Image Concerns

The existence of body image concerns among female university students was evaluated using the 16-item short form of the Body Shape Questionnaire (BSQ-16) derived from the original BSQ-34 which has been previously validated to be used among university students [41, 42]. This short-form BSQ-16 was used to reduce the respondent burden and to shorten the questionnaire. Each item of the questionnaire was explained clearly by the interviewer. The sum of students' responses to this questionnaire generated a score. According to this score, students were classified into four categories: having no concern with shape (< 38), having mild concern with shape (38–51), having moderate concern with shape (52–66), and marked concern with shape (> 66).

### 2.3. Data Analysis

Frequency tables with percentages, means, and standard deviations were used to describe various sociodemographic characteristics, lifestyle behaviors, anthropometric characteristics, dietary habits, and body image concern categories of the study participants. To compare the proportion and means between the study groups, the chi-squared test and Student's *t*-test were employed, respectively. Multiple stepwise regression analysis was applied to detect the factors associated with body shape concerns. All analysis was two-tailed and a *p* value of < 0.05 was considered statistically significant. The Statistical Package for Social Sciences (Version 21, Armonk, NY, USA) was used for data analysis. The datasets used and/or analyzed during the current study are available from the corresponding author upon reasonable request.

## 3. Results

A total of 634 were included in the final analysis, 338 from Egypt and 296 from Lebanon.  Table 1 presents the socioeconomic backgrounds of the Egyptian and Lebanese female university students participating in the study. There are notable significant differences in age, parental education levels, crowding index, and cumulative GPA between the two groups. Lebanese students tend to be slightly older, have parents with higher education levels, live in more crowded conditions, and have a slightly higher cumulative GPA.

Table 2 highlights the dietary habits, lifestyle choices, and perceptions related to body image and eating behaviors among the participants. There are significant differences in the number of meals per day, snacking frequency, breakfast consumption, stress eating, body weight perception, screen time, evaluation of the current diet, physical activity levels, and smoking between the two groups. The table demonstrates that Lebanese students tend to have better perceptions of body shape, spend less screen time, evaluate their diet as less fatty, engage in more physical activity, and engage more in habitual smoking.

Table 3 presents anthropometric data (weight, height, WC, and BMI) and body image scores for the participants. The statistical results present significant differences in height, WC, BMI categories, and body image concerns between the two groups. Lebanese students tend to be taller, have a larger WC, and are more likely to be classified as underweight. Lebanese students report higher levels of body image concerns, with a greater proportion experiencing marked concerns.

Table 4 demonstrates data on WC and BMI categories about body image scores (ranging from no concern to marked concern) among Egyptian and Lebanese participants. Body image concerns are significantly linked to higher WC and BMI. The *p* values for WC and BMI are all < 0.001, indicating statistically significant differences across body image score categories for both nationalities.

Table 5 displays the results of regression analysis using a stepwise approach to examine factors influencing body image concerns among Egyptian and Lebanese female university students.

The table illustrates that in both countries, an enlarged WC is significantly associated with higher body image concerns. The table also demonstrates that having an obese parent is associated with higher body image concerns in both Egyptian and Lebanese university females. In addition, regression analysis detected that ever following a weight loss diet is linked to higher body image concerns among Egyptian university females compared to dieting during the past year for Lebanese university females. However, regular breakfast consumption and a higher number of meals per day are associated with higher body image concerns among the Egyptian group but were not detected among the Lebanese group. An additional significant factor observed among the Egyptian group only was being employed to be linked to higher body image concerns.

## 4. Discussion

Emotional well-being and mental well-being are crucial for maintaining overall good health and lifelong achievements in young adults [43]. The present study attempted to broaden the understanding of the recent trends of the interplay of factors influencing body image such as sociodemographic factors, dietary habits, physical activity, and weight status, which as far as known has not been studied collectively among the female Egyptian and Lebanese university population. Alarmingly, the current study revealed that almost half of the studied sample (46.7% and 48% of the Egyptian and Lebanese university females, respectively) has body image concerns. These results were in accordance with previously reported body image concerns among Lebanese female university students and Egyptian medical students [44, 45]. However, the current prevalence was higher than the data reported for female university students from other five Arab countries [2], which showed that about one-third of the female university students were found to be dissatisfied with their body shape. Conversely, about two-thirds of Brazilian female university students were dissatisfied with their body status [17]. The differences encountered in the prevalence of body shape concerns across countries may be attributed to the diversity of sociocultural factors and health awareness in the studied samples.

A comparison of the level of concern between the Egyptian and Lebanese university females detected a statistically significant difference between the 2 studied groups (*p*=0.009) such that 10% of the whole studied sample of the Lebanese group showed marked body image concern compared to only 3.6% of the Egyptian group. Furthermore, 8.6% versus only 1.2% of the normal-weight Lebanese and Egyptian university females, respectively, were categorized as having marked body image concerns. This, in turn, may suggest that Lebanese university females are more prone to the “internalization of the thin–ideal standard of female beauty” concept previously described by Dittmar et al. in 2004 which substantially contributes to body image dissatisfaction through moderation of media messages of the Western thin ideal female [46, 47].

The association between overweight status, body weight perception, and body image concerns among youth has received much research interest throughout the past years [16, 19, 25, 46]. Most of the studies examining the relationship between overweight/obesity and body image concerns among female university students in a diversity of some Arab and other developing countries indicated that increased weight was associated negatively with body image satisfaction [2, 18–20, 45, 47] with only a few exceptions [48]. A study conducted among female university students in Kuwait reported that 81% of obese females were dissatisfied with their current weight compared to 30% of nonobese females [49]. These data are in accordance to our findings which show that 79.7% and 72% of overweight/obese female students in Egypt and Lebanon, respectively, have body image concerns. Furthermore, a study among female university students in Brazil reported that overweight and obese female students had a 4.5 and 6.7 times higher prevalence, respectively, than those with low weight [17]. In line with these findings, the results of the stepwise regression analysis in the present study revealed that enlarged WC is a significant predictor of increasing body image dissatisfaction scores in both the Egyptian and Lebanese female groups. This unique finding of the current study may reflect the intensified desire to have slim WC to simulate media figures independent of weight status in both the population groups. Reported data suggested that discordance between perceived body image and the desired ideal body shape could initiate negative self-evaluation and result in body image dissatisfaction [30, 50]. This has been clearly shown by data analysis using a regression model which revealed a significant positive association between perceived body image and body image concern score among the Egyptian group through whom overweight and obesity constitute a significantly higher proportion compared to the Lebanese group. These findings in both populations imply that any deviation from what is perceived as the ideal body shape could trigger the development of body image concerns in this group of vulnerable university females.

Several studies have examined the relationship between sociodemographic characteristics and body image concerns among university students with resulting controversial findings [16–19, 26]. The findings of the current study have revealed some important significant associations between body image concerns and some independent sociodemographic, environmental, and dietary behaviors among both Egyptian and Lebanese female university students. A comparison of the sociodemographic characteristics of the Egyptian and Lebanese female students showed some discrepancies that merit noting. The disparities in parental education and crowding index could reflect broader socioeconomic differences between the two population groups. The higher cumulative GPA among Lebanese students might indicate differences in academic pressures or educational systems. All these factors may interplay distinctively in portraying self-body image perception in each specified population.

In the present study, parental obesity using a stepwise regression model was observed to be significantly associated with increasing body image concern score and this was in agreement with the findings of Egyptian medical students in Tanta [45]. Substantial research evidence has documented the positive association between higher BMI and body image dissatisfaction in middle-aged women [51, 52]. In 2018, Handford and colleagues through an experimental study approach examined the impact of negative maternal modeling by criticizing their own body shape on the presence of their preadolescence daughters' and reported a higher body dissatisfaction among the experimental group [53]. Taken all together, this may explain how obese parents, who are dissatisfied with their own body image, may negatively affect the body image satisfaction of their daughters [54].

Analysis of the present data using a regression model clarified a significant positive association between body image concern score and employment status among the Egyptian study group. This positive association between employment status and body image concerns could be considered one of the notable findings of the present study which pinpoints the negative effect of the psychosocial pressure in the work field on this group of young females. This psychosocial pressure and stress may be related to linking thinness to being successful and competent while linking overweight to a lack of self-control and laziness adopted from Westernized cultures [55].

The engagement of university students in unfavorable health-related behaviors such as restrictive dieting and irregular eating patterns has been reported as an emerging trend [56–58]. This trend has been displayed clearly in the present study as a relatively overall high prevalence of dieting for weight control purposes at 40.2% and 42.7% among Egyptian and Lebanese groups of the present sample, respectively. This dieting behavior is higher than the data reported in 2012 by female university students from Bahrain, Egypt, Oman, Syria, and Jordan, which showed that 22%–37% of the participants dieted to lose weight (2), but lower than the dieting prevalence of medical Egyptian students reported in 2015. The reported variation in the prevalence of dieting behavior could be attributed to differences related to the field of study, socioeconomic and cultural factors between these countries, as well as the rapidly changing beauty ideal throughout the past few years. Furthermore, the results of regression analysis showed that previous dieting trials and dieting in the last year were significantly related to higher body image concerns in the Egyptian and Lebanese groups, respectively. Some authors have declared that restricting food among university females usually begins as a reaction to the nonacceptance of pubertal body changes and weight gain and in response to the strong sociocultural pressure of attaining a slim appearance [59]. Conversely, exploration of the relationship between meal patterns and body image concern revealed a statistically significant positive association between a higher number of meals and regular intake of breakfast among the Egyptian group only. Given that the Egyptian group reported a higher intake of fatty meals and exhibited poorer self-perception of body shape than the Lebanese group (Table 2), the latter mentioned that this significant association could be explained by the findings of Ribeiro-Silva and colleagues who hypothesized that poor self-perception of body shape coupled with body image dissatisfaction among the youth may result in overeating after failed trials of dietary restriction [60]. This hypothesis was further corroborated by the findings among physical education students in Australia, who showed decreased body image satisfaction after intake of unhealthy breakfast meals [61].

## 5. Conclusion

The present study highlights new evolving determinants of body image concerns development with some concordance among Egyptian and Lebanese university female students most notably abdominal obesity which was found to be the strongest determinant. Conversely, some other differences in the determinants of body image concerns such as perceived body image, meal pattern, and employment were detected among Egyptian university females only. These important significant findings may serve as a milestone for tailoring the most relevant culture-specific preventive programs, aimed at raising health awareness and improving body image satisfaction.

## 6. Study Limitations

The cross-sectional study design in the present study does not allow disclosing the trends of changes in body image concerns and related determinants. In addition, the study sample did not include students from public universities which usually enroll students mainly from lower or lower-middle classes and included students enrolled in two private universities which represent the lower–upper middle social class which makes it difficult for data generalization to the whole female university population in both countries. The relatively large sample size of the present study which included 634 students and the lack of funding made it difficult to perform detailed body composition analysis using one of the validated techniques such as DEXA or bioelectrical impedance analysis, which if applied would have contributed to a better understanding of the significance of body fat mass or its distribution on the development of body image concerns; however, we only applied the use of BMI and WC, which could be considered one of the additional shortcomings of the present study.

## Figures and Tables

**Table 1:** Sociodemographic characteristics of the study population distributed according to nationality.

	Nationality		*p* value
	Egypt	Lebanon	
Age	20.4 ± 1.7	21.0 ± 2.0	< 0.001^∗∗^
Marital status	*N* (%)	*N* (%)	0.838^∗∗∗^
Single	322 (95.3)	280 (94.9)	
Married	16 (4.7)	16 (5.1)	
Father's education			
Illiterate	6 (1.8)	7 (2.4)	0.005^∗∗∗^
Primary	66 (19.5)	32 (10.8)	
Vocational	14 (4.1)	22 (7.4)	
Secondary	88 (26.0)	63 (21.6)	
University	164 (48.5)	171 (57.8)	
Mother's education			
Illiterate	6 (1.8)	6 (2.0)	0.010^∗∗∗^
Primary	50 (14.5)	50 (16.9)	
Preparatory	8 (2.4)	17 (5.8)	
Secondary	142 (42.0)	88 (29.7)	
University	132 (39.1)	135 (45.6)	
Area of residence			
Urban	270 (79.9)	239 (80.8)	0.731^∗∗∗^
Rural	68 (20.1)	57 (19.2)	
Parental obesity			
Yes	100 (29.6%)	80 (27.0%)	0.480^∗∗∗^
No	238 (70.4%)	216 (73.0%)	
History of parental obesity–related illness			
No	52 (15.4%)	46 (15.4%)	0.999^∗∗∗^
Yes	286 (84.6%)	250 (84.6%)	
Cumulative GPA	2.9 ± 0.55	3.02 ± 0.47	0.046^∗∗∗^
Crowding index			
Less than 1	192 (56.8%)	157 (53.1%)	0.001
[1-2]	137 (40.6%)	110 (37.2%)	
2 or more	9 (2.6%)	29 (9.7%)	
Employment			
Employed	52 (15.4%)	33 (11.2%)	0.121
Unemployed	286 (84.6%)	263 (88.8%)	

*Note:* Data are presented as mean ± standard deviation (SD) or frequency (percentage) as appropriate.

^∗∗^ *p* value based on *t-*tests for comparison of two means with values less than 5% is considered significant.

^∗∗∗^ *p* value based on chi-square tests to compare proportions with values less than 5% is considered significant.

**Table 2:** Dietary and lifestyle characteristics of the study population distributed according to nationality.

	Nationality		*p* value
	Egypt (*n* = 338) *N*. %	Lebanon (*n* = 296) *N*. %	
Number of meals/day			
One	18 (5.3%)	9 (3.0%)	0.03^∗∗^
Two	118 (34.9%)	104 (35.1%)	
Three	184 (54.4%)	150 (50.8%)	
Four or more	18 (5.3%)	33 (11.1%)	
Snacks per day			
Zero	0 (0.0%)	3 (1.0%)	0.17^∗∗^
One	66 (19.5%)	48 (16.2%)	
Two	124 (36.7%)	114 (38.5%)	
Three	92 (27.2%)	71 (24.0%)	
Four or more	56 (16.6%)	60 (20.3%)	
Do you eat breakfast?			
Never	14 (4.1%)	13 (4.4%)	0.003^∗∗^
Regularly	218 (64.5%)	153 (51.6%)	
Sometimes	106 (31.4%)	130 (44.0%)	
Have you ever been on a diet?			
No	202 (59.8%)	170 (57.4%)	0.528^∗∗^
Yes	136 (40.2%)	126 (42.6%)	
Have you ever been on a diet last year?			
No	220 (65.1%)	199 (67.3%)	0.549^∗∗^
Yes	118 (34.9%)	97 (32.7%)	
What motivates you to eat healthy?			
Social norms	106 (31.4%)	81 (27.4%)	0.271^∗∗^
Health norms	232 (68.6%)	215 (72.6%)	
Do you eat more when you are stressed preparing for your exams?			
No	142 (42.0%)	76 (25.7%)	< 0.001^∗∗^
Rarely	6 (1.8%)	16 (5.4%)	
Sometimes	68 (20.1%)	71 (24.0%)	
Yes	122 (36.1%)	133 (44.9%)	
How do you perceive your current body weight?			
Underweight	18 (5.3%)	13 (4.4%)	0.009^∗∗^
Normal	204 (60.4%)	213 (71.9%)	
Overweight	112 (33.1%)	64 (21.7%)	
Obese	4 (1.2%)	6 (2.0%)	
Screen time			
1 h-2 h	93 (27.5%)	99 (33.4%)	0.002^∗∗^
3 h-4 h	71 (21.0%)	89 (30.2%)	
5 h-6 h	71 (21.0%)	52 (17.6%)	
7 h or more	103 (30.5%)	56 (18.8%)	
Do you eat to comfort yourself?			
Yes	206 (60.9%)	173 (58.4%)	0.520^∗∗^
No	132 (39.1%)	123 (41.6%)	
Do you eat while watching TV?			
Never	122 (36.1%)	91 (31.1%)	
Yes	216 (63.9%)	204 (68.9%)	0.182^∗∗^
How do you evaluate your current diet?			
Adequate	172 (50.9%)	151 (51.1%)	0.007^∗∗^
Too much sugar	66 (19.5%)	70 (23.6%)	
Too much fat	76 (22.5%)	40 (13.5%)	
Not enough	24 (7.1%)	35 (11.8%)	
Physical activity score (IPAQ)			
Inactive	72 (21.3%)	91 (30.4%)	0.002^∗∗^
Minimally active	232 (68.6%)	163 (55.1%)	
Active	34 (10.1%)	43 (14.5%)	
Smoking			
Nonsmoker	284 (84.0%)	214 (72.2%)	0.001^∗∗^
Occasional	28 (8.3%)	35 (11.9%)	
Smoker	26 (7.7%)	47 (15.9%)	

*Note:* Data are presented as mean ± standard deviation (SD) or frequency (percentage) as appropriate.

^∗∗^ *p* value based on chi-square tests with values less than 5% is considered significant.

**Table 3:** Anthropometric characteristics and body image score among the study population distributed according to nationality.

	Nationality		*p* value
	Egypt	Lebanon	
Weight	60.8 ± 10.4	59.8 ± 9.9	0.208^∗∗^
Height	161.4 ± 6.8	163.4 ± 6.1	< 0.001^∗∗^
WC	73.4 ± 10.1	83.0 ± 10.9	< 0.001^∗∗^
^∗^BMI	*N*. %	*N*. %	
Underweight	24 (7.1%)	73 (24.3%)	< 0.001^∗∗∗^
Normal	208 (61.5%)	174 (58.7%)	
Overweight/obese	106 (31.4%)	49 (17.0%)	
Body image score			
No concern	180 (53.3%)	154 (52.0%)	0.009^∗∗∗^
Mild concern	90 (26.6%)	70 (24.0%)	
Moderate concern	56 (16.6%)	41 (13.9%)	
Marked concern	12 (3.6%)	31 (10.1%)	

^∗^BMI classification is based on the WHO criteria [36].

^∗∗^ *p* value based on *t-*tests for comparison of the two means with values less than 5% is considered significant.

^∗∗∗^ *p* value based on chi-square tests to compare proportions with values less than 5% is considered significant.

**Table 4:** Anthropometric characteristics of the study population distributed according to nationality and body image score.

		No concern	Mild	Moderate	Marked concern	*p* value
^∗^BMI		*N*. %	*N*. %	*N*. %	*N*. %	
Underweight	Egyptian (*n*. = 338)	52 (89.7%)	6 (10.3%)	0 (0%)	0 (0%)	< 0.001^∗∗∗^
Normal		106 (61.6%)	46 (26.7%)	18 (10.5%)	2 (1.2%)	
Overweight/obese		22 (20.4%)	38 (35.2%)	38 (35.2%)	10 (9.3%)	
Underweight	Lebanese (*n*. = 296)	56 (77.8%)	7 (9.7%)	6 (8.3%)	3 (4.2%)	< 0.001^∗∗∗^
Normal		84 (48.3%)	51 (29.3%)	24 (13.8%)	15 (8.6%)	
Overweight/obese		14 (28%)	13 (26%)	11 (22%)	12 (24%)	
^∗∗^WC						
Normal	Egyptian (*n*. = 338)	156 (62.9%)	58 (23.4%)	26 (10.5%)	8 (2.4%)	< 0.001^∗∗∗^
Enlarged		24 (26.7%)	32 (35.6%)	30 (33.3%)	4 (4.4%)	< 0.001^∗∗∗^
Normal	Lebanese (*n*. = 296)	68 (63.6%)	21 (19.7%)	12 (11.1%)	6 (5.6%)	0.008^∗∗∗^
Enlarged		82 (43.4%)	50 (26.5%)	27 (14.2%)	30 (15.8%)	< 0.001^∗∗∗^

^∗^BMI classification is based on the WHO criteria [36].

^∗∗^WC is the enlarged waist circumference based on the WHO criteria [37].

^∗∗∗^ *p* value based on chi-square tests to compare proportions with values less than 5% is considered significant.

**Table 5:** Body image concerns' determinants among the Egyptian and Lebanese university female students.

	Egypt			Lebanon		
	Coefficient	*t-*test	*p* value^∗∗^	Coefficient	*t-*test	*p* value^∗∗^
Constant	14.560	1.703	0.090	37.957	2.533	0.013
Enlarged WC	0.310	4.068	≤ 0.001	0.492	3.533	≤ 0.001
Obesity of one parent	3.557	2.333	0.020	10.575	3.133	0.002
Ever been on a diet before	9.494	6.094	≤ 0.001			
Dieting during the past year				11.313	3.490	≤ 0.001
Perceived increased body weight	7.553	5.587	≤ 0.001			
Regular intake of breakfast	3.835	2.897	0.004			
Number of meals per day	2.515	2.512	0.013			
Employment	5.878	−3.140	0.002			

*Note:* Dependent variable: body image score.

^∗∗^ *p* value based on *t-*tests for comparison of the two means with values less than 5% is considered significant.

## Data Availability

The data that support the findings of this study are available from the corresponding author upon reasonable request.
